# Retraction: Homocysteinylated Albumin Promotes Increased Monocyte-Endothelial Cell Adhesion and Up-Regulation of MCP1, Hsp60 and ADAM17

**DOI:** 10.1371/journal.pone.0215587

**Published:** 2019-04-12

**Authors:** The PLOS ONE Editors

Following publication of this article [1], the following concerns were noted:

- Fig 4A GAPDH panel lanes 1–7 appear highly similar; there are vertical discontinuities between lanes 3 and 4.
- Fig 7B MCP1 panel appears to contain vertical discontinuities between lanes 1, 2 and lanes 2, 3, as well as a horizontal discontinuity beneath the band in lane 1.
- Fig 7B MCP1 panel lane 2 appears similar to ICAM1 panel lane 1 when horizontally flipped and stretched.
- Fig 7B actin panel all lanes appear to have similar dotted patterns around the bands.

For Fig 7B the authors were not able to provide raw images of all full gels. The authors commented that Fig 4A lanes 1–3 and lanes 4–7 represent separate gels and a composite image was generated for the purposes of figure presentation. The underlying images provided do not resolve all concerns noted.

The authors have explained that the primary data underlying other experiments except for microarray data are not available.

In light of the above-mentioned image concerns and the unavailability of underlying data, the *PLOS ONE* Editors retract the article.

AFP and DI did not agree with the retraction. DLP responded but did not provide a comment on the retraction. RC, IS, AC, SS, CL, FA, and EA did not respond.
